# Detection of Cephalosporin and Fluoroquinolone Resistance Genes via Novel Multiplex qPCR in Fecal *Salmonella* Isolates From Northern Californian Dairy Cattle, 2002–2016

**DOI:** 10.3389/fmicb.2021.601924

**Published:** 2021-02-15

**Authors:** Carl Basbas, Barbara A. Byrne, Munashe Chigerwe, Edlin D. Escobar, Emir Hodzic, Alda F. A. Pires, Richard V. Pereira

**Affiliations:** ^1^Department of Population Health and Reproduction, School of Veterinary Medicine, University of California, Davis, Davis, CA, United States; ^2^Department of Pathology, Microbiology and Immunology, School of Veterinary Medicine, University of California, Davis, Davis, CA, United States; ^3^Department of Medicine and Epidemiology, School of Veterinary Medicine, University of California, Davis, Davis, CA, United States; ^4^Real-Time PCR Research and Diagnostics Core Facility, School of Veterinary Medicine, University of California, Davis, Davis, CA, United States

**Keywords:** fluoroquinolone, cephalosporin, qPCR, antimicrobial resistance genes, *Salmonella*

## Abstract

The objectives of this study were to evaluate the prevalence of extended spectrum β-lactamase (ESBL) genes, AmpC-type β-lactamase (ACBL) genes, and plasmid mediated quinolone resistance (PMQR) genes in *Salmonella* isolated at a Veterinary Medical Teaching Hospital microbiology laboratory, examine trends in presence of these resistance genes, and to explore the correlation between phenotypic resistance and presence of specific genes. The presence of ESBL, ACBL, and PMQR genes were detected using a single, novel multiplex qPCR. Only the genes *bla*_CMY–2_ and *bla*_TEM_ were detected in the 110 *Salmonella* isolates tested. PMQR genes were not detected in isolates screened. Of 94 third-generation cephalosporin resistant isolates, representing eight serotypes, 48% (*n* = 45) were positive for *bla*_CMY–2_ only and 50% (*n* = 47) were simultaneously positive for *bla*_CMY–2_ and *bla*_TEM_. Two third-generation cephalosporin resistant isolates were tested negative for all β-lactamase genes in our qPCR assay and likely house ESBL genes not screened for by our qPCR assay. A logistic regression model revealed that for serotype Dublin isolates (*n* = 38) the odds ratio for testing positive for *bla*_TEM_ when compared to all other serotypes was 51.6 (95% CI: 4.01–664.03, *p* = 0.0029). For serotype Typhimurium (*n* = 9) the odds ratio for testing positive for *bla*_TEM_ when compared to all other serotypes was 43.3 (95% CI: 1.76–1000, *p* = 0.0216). Overall, our results suggest that the prevalence of resistance to cephalosporins and fluoroquinolones due to ESBLs, ACBLs, and PMQR genes present in bovine nontyphoidal *Salmonella enterica* isolates has remained relatively constant in the isolates screened over a 14-year period.

## Introduction

Globally in 2017, around 91 million cases of human gastrointestinal illness and diarrhea were believed to be caused by nontyphoidal *Salmonella enterica* (**NTS**) (Stanaway et al., 2019). In the United States alone, 1.35 million NTS infections amounted to an estimated $400 million in medical costs annually (Centers for Disease Control and Prevention, 2019). In humans, severe infections caused by *Salmonella* usually require treatment with specific recommended antimicrobials, including ciprofloxacin, azithromycin, and ceftriaxone (Centers for Disease Control and Prevention, 2019). With 3% and 7% of all human NTS infections in the U.S. classified as either ceftriaxone resistant or ciprofloxacin nonsusceptible, respectively, the U.S. Center for Disease Control and Prevention (CDC) has designated drug resistant NTS as a serious threat (Centers for Disease Control and Prevention, 2019). As resistance to third-generation cephalosporins and fluoroquinolones grows, increasing attention is being placed on extended spectrum β-lactamase (ESBL) genes, AmpC-type β-lactamase (ACBL) genes, and plasmid mediated quinolone resistance (PMQR) genes.

Extended spectrum β-lactamase (ESBL) genes encode for enzymes which are able to cleave the β-lactam ring of a wide range of β-lactam antimicrobials (e.g., penicillins and cephalosporins) (Dhillon and Clark, 2012). They confer β-lactam resistance to the bacteria that produce them, primarily *Klebsiella pneumoniae* and *Escherichia coli*. Worldwide, the most common ESBLs are the SHV, TEM, and CTX-M types. AmpC-type β-lactamase (ACBL) genes also encode for enzymes capable of degrading β-lactam antibiotics, including: extended spectrum cephalosporins (excluding cefepime and cefpirome), cephamycins, and ceftriaxone (Jacoby, 2009; Polsfuss et al., 2011). *bla*_CMY–2_ is the most common plasmid mediated ACBL gene globally (Jacoby, 2009).

Resistance to the quinolone and fluoroquinolone classes of antimicrobials has generally been attributed to chromosomal mutations in the bacterial enzymes targeted by these classes of antimicrobials: DNA gyrase and DNA topoisomerase IV (Strahilevitz et al., 2009). Additionally, three types of plasmid mediated quinolone resistance (PMQR) mechanisms have been identified: *qnr* genes protect DNA gyrase, the *aac(6′)-lb-cr* gene acetylates ciprofloxacin, norfloxacin, and certain other quinolones, and *oqxAB* and *qepA* genes produce efflux pumps (Rodriguez-Martinez et al., 2016; Karp et al., 2018).

Currently, a common method for identification of ESBL-producing bacteria is via culture-based phenotypic methods (Clinical and Laboratory Standards Institute, 2011; The European Committee on Antimicrobial Susceptibility Testing, 2013). Unfortunately, a definitive negative result may take 24–120 h (Engel et al., 2017). Furthermore, as these methods depend on the inhibition of ESBLs by clavulanic acid, the production of additional AmpC or metallo-β-lactamases (which are uninhibited by clavulanic acid) may decrease test sensitivity (Rawat and Nair, 2010). To identify particular genes responsible for ESBL production, reference laboratories use molecular analyses, primarily polymerase chain reaction (PCR) (Wittum et al., 2012).

Detection of ACBL-producing bacteria is generally done using phenotypic tests utilizing ACBL inhibitors such as boronic acid and cloxacillin, however, these tests are unable to distinguish between chromosomal or plasmid-mediated AmpC-type β-lactamases (Tamma et al., 2019). Detection of isolates carrying plasmid-mediated ACBL genes may be particularly important as these isolates may appear to be susceptible to cephalosporins *in vitro*, only to fail to respond to treatment (Thomson, 2001). Molecular approaches to identify plasmid-mediated ACBL genes are available, but are typically unavailable in clinical laboratories (Perez-Perez and Hanson, 2002). Additionally, there is currently no Clinical and Laboratory Standards Institute (CLSI) test for AmpC-type β-lactamases in clinical isolates (Clinical and Laboratory Standards Institute, 2020).

While there are multiplex PCR methods available for the detection of either ESBL, ACBL, or PMQR genes, there are few published multiplex PCR methods available for the combined detection of ESBL, ACBL, and PMQR genes relevant to NTS treatment (Ciesielczuk et al., 2013; Roschanski et al., 2014). The goal of this study was to identify trends in resistance of fecal *Salmonella* isolates to cephalosporins and fluoroquinolones due to the presence of ESBL, ACBL, and PMQR genes from *Salmonella* isolates obtained from cattle fecal samples isolated and tested in the University of California, Davis William R. Pritchard Veterinary Medical Teaching Hospital (VMTH) microbiology laboratory during a 14-year interval using a single, novel multiplex qPCR method.

## Materials and Methods

### Isolate Collection and Selection

A total of 110 *Salmonella* isolates were selected for qPCR analysis from a collection of 242 *Salmonella* isolates recovered from 9162 bovine fecal samples submitted to the University of California, Davis William R. Pritchard VMTH microbiology laboratory between January 1, 2002 and December 31, 2016 as detailed previously (Davidson et al., 2018). Sixty-eight isolates were recovered from dairy cattle exhibiting clinical signs of *Salmonella* infection, while 42 isolates were recovered from asymptomatic dairy cattle through the VMTH Infectious Disease Control (IDC) program. All isolates with phenotypic resistance to at least one of the following drugs, nalidixic acid, ceftiofur, and/or ceftriaxone, were included in the study (*n* = 94; Supplementary Table S1). None of the isolates were phenotypically resistant to ciprofloxacin (MIC ≥ 1.0 μg/ml). Only two isolates were resistant to nalidixic acid, and also presented simultaneous phenotypic resistance to ceftriaxone and ceftiofur. All isolates phenotypically resistant to ceftiofur were also resistant to ceftriaxone.

For each year a pan-susceptible *Salmonella* isolate, when available, was selected to serve as a control (*n* = 16) (Supplementary Table S2). For two years, namely 2002 and 2004, no pan-susceptible isolates were available, and an isolate resistant to streptomycin, and one isolate resistant to ampicillin, streptomycin, and tetracycline, respectively, were selected (Supplementary Table S2). These isolates were selected on the criteria that they were susceptible to quinolone and cephalosporin drugs and were the isolates resistant to the fewest number of antimicrobials for that year.

### Antimicrobial Susceptibility Testing

Data from phenotypic antimicrobial susceptibility testing conducted on the same isolates from a previous study were used (Davidson et al., 2018). Briefly, for that study all isolates were tested using the standardized National Antimicrobial Resistance Monitoring System (NARMS) (Thermo Fisher, Sensititre CMV3AGNF) for aerobic Gram-negative bacteria that included penicillins (ampicillin), β-lactam/ β-lactamase inhibitor combinations (amoxicillin/clavulanic acid), cephalosporins (ceftriaxone, ceftiofur, and cefoxitin), quinolones (ciprofloxacin and nalidixic acid), phenicols (chloramphenicol), sulfa-based drugs (sulfisoxazole and sulfamethoxazole/trimethoprim), tetracyclines (tetracycline), macrolides (azithromycin), and aminoglycosides (gentamicin and streptomycin). Plates were read using the Sensititre Vizion System^®^ (Thermo Fisher) and minimum inhibitory concentrations (MIC) were interpreted using NARMS breakpoints (Centers for Disease Control and Prevention, 2014).

### DNA Extraction

Frozen isolates were streak plated on blood agar plates and incubated overnight at 37°C to check for contamination. Visual inspection did not show any contamination therefore 1.5 ml of autoclaved BHI broth in a 2 ml micro centrifuge tube was inoculated from each isolate in a biological safety cabinet. The DNA was then extracted according to the manufacturer’s instructions for the DNeasy Blood and Tissue Kit (Qiagen N.V., Carlsbad, CA, United States). Two hundred microliter of DNA was eluted for each isolate into a sterile 2 ml micro centrifuge tube. The DNA samples were then stored at −80°C until further downstream processing.

### Multiplex qPCR Development and Validation

In collaboration with the UC Davis Real-time PCR Research and Diagnostics Core Facility, a singleplex and several multiplex (duplex and triplex) qPCR assays were developed to facilitate rapid and sensitive analysis of samples. Isolates were analyzed for the presence of β-lactamase encoding genes (*bla*_TEM_, *bla*_CTX–M_, and *bla*_CMY–2_) and for presence of plasmid mediated quinolone resistance (PMQR) genes (*oqx*A, *oqx*B, *qnr*S, *qnr*B, and *aac(6′)-Ib-cr*). q*n*rA was not included in the qPCR assay because it has been rarely identified in *Salmonella* isolates with phenotypic resistance to fluoroquinolones. Cattle studies screening for *qnr* genes have more frequently detected *qnr*B and *qnr*S (Carroll et al., 2017). Furthermore, other recent studies screening *Salmonella* from isolates originating from broiler chicken and pork products for *qnr* genes did not detect *qnr*A genes (Tyson et al., 2017; Mahmud et al., 2018). Other recent studies screening human *Salmonella* isolates have also not detected *qnr*A, and noted it as infrequently detected when compared to *qnr*B and *qnr*S (Carroll et al., 2017; Karp et al., 2018).

Sequences from GenBank (*bla*_TEM_ (LT985387), *bla*_CTX–M_ (CP025146), *bla*_CMY–2_ (KY612500), *oqx*A (CP019074), *oqx*B (CP019074), *qnr*S (CP026578), *qnr*B (KP012539), and *aac(6′)-Ib-cr* (NG_056043)) were aligned using Sequence Analysis and Molecular Biology Data Management software Vector NTI AdvanceTM11 (Thermo Fisher Scientific, Carlsbad, CA, United States). The alignment was used to design primers specific to target for singleplex and multiplex qPCRs assays (Table 1). The specificity of the primers and probes was confirmed by BLAST searching against the non-redundant database of GenBank (NCBI). The primers for detecting *bla*_TEM_ in our qPCR assay were designed to be more general and capable of annealing to both TEM non-extended spectrum β-lactamases and TEM-type ESBLs. This was done in the context of our assay being used as a screening tool.

**TABLE 1:** Primer and probe sequences for qPCR assays.

**Primers/probe**	**Sequence 5′–3′**	**Amplicon size (bp) and %GC**
*bla*_TEM_-97f	GATGCTGAAGATCAGTTGGGTG	71 bp, 50.7%
*bla*_TEM_-168r	CTCAAGGATCTTACCGCTGTTGA	
*bla*_TEM_-123p	FAM-AGTGGGTTACATCGAAC MGB	
*oqx*A-1079f	ATAGCGTCATCGTCGACGG	73 bp, 49.3%
*oqx*A-1152r	CATGGCAACGGTTTTGGC	
*oqx*A-1114p	VIC-ATGCCGGGTATGCC-MGB	
*qnr*S-523f	GTTGACGAATGTCGTATCACGC	73 bp, 50.1%
*qnr*S-596r	TCACCTTCACCGCTTGCAC	
*qnr*S-553p	TET-ACGTCGAAAGTCGCTG-MGB	
*bla*_CTX–M_-792f	TTACTTCACCCAGCCTCAACCT	59 bp, 57.6%
*bla*_CTX–M_-851r	GCCGCCGACGCTAATACA	
*bla*_CTX–M_-816p	FAM-GGCAGAAAGCCGTCG-MGB	
*bla*_CMY–2_-884f	CCGATATCGTTAATCGCACCAT	63 bp, 55.5%
*bla*_CMY–2_-947r	ACGGCCATACCCGGAATAG	
*bla*_CMY–2_-911p	VIC-CGTTGATGCAGGAGC-MGB	
*oqx*B-1361f	TTCCGTCCGTTTAACCGCT	61 bp, 55.7%
*oqx*B-1422r	TTGCCTACCAGTCCCTGATAGC	
*oqx*B-1385p	TET-CTGCGCAGCTCGAA-MGB	
*aac*6-lb-59f	GCGATGCTCTATGAGTGGCTAA	73 bp, 56.1%
*aac*6-lb-132r	AGTGTCGGGCGTGCTTCTT	
*aac*6-lb-90p	FAM-ATATCGTCGAGTGGTGGG-MGB	
*qnr*B-276f	TTCAGATCTCTCCGGCGG	72 bp, 54.2%
*qnr*B-348r	GGTCAGATCGCAATGTGTGAAG	
*qnr*B-304p	VIC-ACTTTCGACTGGCGAGC-MGB	

*Fluorophores (FAM, TET, VIC) were specific to each probe, and amplicon lengths are also provided.*

Different fluorophores for each multiplex: *bla*_TEM_, *bla*_CTX–M_, *aac(6′)-Ib-cr* all used the fluorescent probe 6-carboxy-fluorescein (FAM); *oqx*A, *bla*_CMY–2_, and *qnr*B used the VIC probe; whereas, *qnr*S and *oqx*B used tetrachlorofluorescein (TET). All the probes utilized a 3′ minor groove binding quencher (Table 1). All qPCR assays were designed using Primer Express (Thermo Fisher Scientific) following the guidelines for multiplex qPCR assays. Amplicon lengths were ranging from 59 to 73 bp with each multiplex having similar lengths and GC percentage (Table 1). Primers and probes were synthesized by Life Technologies (Grand Island, NY, United States).

To test the efficiency of each primer/probe combination, singleplex mixes were prepared by combining 20 μL of 100 pmol/μl forward primer, 20 μl of 100 pmol/μl reverse primer, and 4 μl each of the 100 pmol/μl probes individually, in a final volume of 240 μl water. The qPCR multiplex primer/probe mix was prepared by mixing 40 μl for duplex and 60 μl for triplex of 100 pmol/μl forward primer, 40μl for duplex and 60 μl for triplex of 100 pmol/μl reverse primer, and 4 μl each of the two or three 100 pmol/μl probes in a final volume of 240 μl water.

The singleplex and multiplex qPCR for each target contained 0.42 μl water, 0.58 μl primer/probe mix (final concentration 400 nM of each primer and 80 nM probe), 6 μl of commercially available TaqMan^TM^ Universal Master Mix (UMM) (Thermo Fisher Scientific) for singleplex or Gene Expression Master Mix (Qiagen) for multiplex, and 5 μl of the DNA in a final volume of 12 μl.

All samples were placed in a 384-well plate and amplified in a 7900HT FAST Real-time PCR system (Thermo Fisher Scientific) using the manufacturer’s standard amplification conditions (2 min at 50°C, 10 min at 95°C, then 40 cycles of 15 s at 95°C and 60 s at 60°C). Fluorescent signals were collected during annealing and quantitative cycle (Cq) was calculated and exported with a threshold of 0.15 and a baseline of 3–10 for FAM labeled assays, 0.20 and a baseline of 3–10 for VIC assays, and 0.10 and a baseline of 3–15 for TET assays. The *C*_q_ was defined as the cycle in which there was a significant increase in reporter signal of the amount of PCR product detected during the exponential phase, above the threshold.

### qPCR Assay Validation

To assess and validate the efficiency of singleplex and multiplex qPCR assays for all assays, endpoint analysis of DNA using 10-fold dilutions was performed for each assay. In the singleplex qPCR mixtures (*qnr*S), only one target positive control was conducted using a nucleic acid template of known copy number. In multiplex qPCR mixtures, each of the two (*bla*_TEM_ and *oqx*A, *aac(6′)-Ib-cr*, and *qnr*B) or three (*bla*_CTX–M_, *bla*_CMY–2_, and *oqx*B) target positive controls was combined in a single amplification tube. Standard curves were generated for each set of 10-fold serial dilutions of target. We calculated the amplification efficiency (E) of all assays from the slope (S) of the standard curves, using the formula *E* = 10^1/–s^ - 1 (Supplementary Table S3).

Sensitivity⁢log⁢(Sl)=(40-y⁢intercept)/S.

$$\mathrm{Sensitivity}\,\mathrm{copy}\,\mathrm{number}\,\left(\mathrm{CN}\right)=10^{\text{Sl}}$$

The multiplexes were very similar in sensitivity when all three targets were compared in the reaction. The sensitivity of each assay run as a single or multiplex was ∼10 or ∼100 gene copies. Such high similarity between the assays’ efficiency, sensitivity, amplicon length, and melting temperature assured the same competition efficiency during multiplex qPCR reaction.

### Statistical Analysis

**FIGURE 1:**
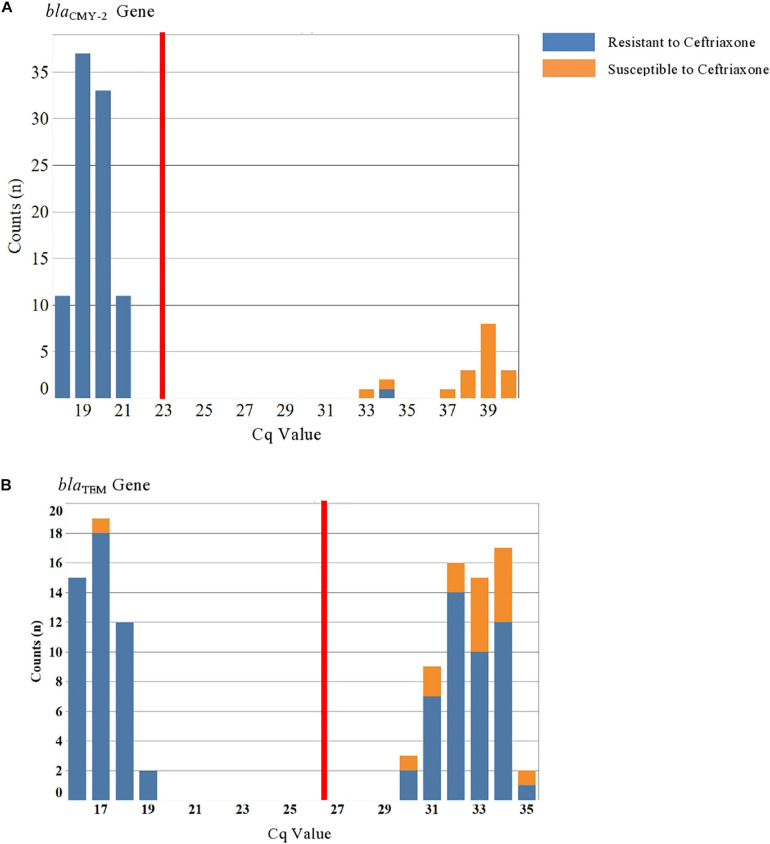
Histogram with cycle quantification (*C*_q_) value distribution for *Salmonella* isolates screened for *bla*_CMY–2_ and *bla*_TEM_ using multiplex qPCR. Blue represents isolates phenotypically resistant to ceftriaxone and orange represents isolates phenotypically susceptible to ceftriaxone. The red line indicates the cut-off *C*_q_ values for *bla*_CMY–2_ and *bla*_TEM_. (A) *bla*_CMY–2_ gene and *bla*_TEM_ gene. **Graph A** depicts the Cq value distribution for blaCMY-2, and **graph B** depicts the Cq value distribution for blaTEM.

Descriptive analysis for the distribution of *Salmonella* by year, phenotype, and resistance genes detected was conducted in JMP (SAS Institute Inc., Cary, NC, United States). To evaluate the reproducibility of *C*_q_ measurements and their associated error of the mean, a histogram was generated to visually evaluate the data (Figure 1) for each gene detected by qPCR, and mean and standard error for *C*_q_ values (Karlen et al., 2007). A 99% confidence interval of the standard error of the mean for *C*_q_ values was used for each gene to select the cut-off value for *C*_q_ values to classify an isolate as being positive for carrying that gene.

Logistic regression models using the GLIMMIX function in SAS using the logit link function were used to evaluate the population of *bla*_CMY–2_ positive, ceftriaxone resistant *Salmonella* isolates (*n* = 92) for the effect of explanatory variables sex, serotype, submission type (IDC vs. Suspect), and year group (calf vs. adult) on the risk of detecting an isolate positive for the gene *bla*_TEM_. Three models were constructed that differed by the presence of a binomial variable that allowed the evaluation of the effect of each of these three serotypes: *S*. Dublin, *S.* Typhimurium, and *S*. Newport when compared against any other serotype. This was a binomial variable that compared one of the three serotypes to all other serotypes combined (e.g., Dublin vs. Typhimurium, Newport, and any other serotype present in the study dataset). These three serotypes were selected because they represented the top three serotypes for isolates selected (92% of all isolates). Year group (2002–2009 vs. 2010–2016) when isolates were collected was included and maintained in all models as an independent variable to evaluate temporal changes on antimicrobial resistance. These two multi-year periods were chosen because they represent two halves of the time period evaluated. Additionally, these time periods were selected because of legislation related to antimicrobial use that occurred after 2009, such as changes on how ceftiofur could be used in an extra-label manner in livestock (Schmidt, 2012). Any explanatory variable that was not significant was removed from the model after evaluating if it negatively affected the model by evaluating the Akaike Information Criterion (AIC) value and overdispersion was evaluated using the Pearson chi-square divided by the degrees of freedom value. For all models, a *P* value of <0.05 was considered a significant difference.

## Results

### Cycle Quantification (*C*_q_) Value Distribution for *bla*_CMY–2_ and *bla*_TEM_ Genes

Figure 1 contains two histograms of the *C*_q_ values for each of the two genes detected in our study: *bla*_CMY–2_ and *bla*_TEM_ (Figures 1A,B). Only two genes were detected in the 110 *Salmonella* isolates tested, namely *bla*_CMY–2_ and *bla*_TEM_. The mean and 99% confidence interval for the *C*_q_ values of 23.0 (99% CI: 21.7–24.2) and 26.4 (99% CI: 24.9–27.7) were determined for *bla*_CMY–2_ and *bla*_TEM_, respectively. Figure 1A, which depicts the *C*_q_ value distribution for *bla*_CMY–2_, indicates that isolates below the cut-off of 23 were phenotypically resistant to ceftriaxone (Figure 1, blue color).

The opposite is primarily also true as most isolates above the cut-off of 23 are phenotypically susceptible to ceftriaxone (Figure 1, orange color). Results for logistic regression evaluating the effect of explanatory variables on the odds of isolating *bla*_CMY–2_-positive, ceftriaxone resistant *Salmonella* isolates also carrying *bla*_TEM_ is depicted in Table 2.

**TABLE 2:** Summary of the logistic regression model evaluating the effect of the explanatory variables serotype, year group, and submissionn type on the odds ratio of isolating a *bla*_CMY–2–_positive, ceftriaxone resistant *Salmonella* isolate also carrying *bla*_TEM_.

**Variable**	**Coefficient**	**SE**	%(*n*)**	**OR**	**OR (95% confidence interval)**		***p* value**
					**Lower**	**Upper**	
Intercept	−2.06	1.38					
**Serotype^1^**							
Dublin	3.94	1.28	89 (38)	51.6	4.01	664.03	**0.0029**
Typhimurium	3.77	1.61	78 (9)	43.3	1.76	1000	**0.0216**
Newport	−0.04	1.21	14 (42)	0.96	0.087	10.5	0.97
**Year Group^2^**							
2002–2009	−0.13	0.84	46 (86)	0.88	0.17	4.66	0.88
2010–2016	Reference*		33 (24)				
**Submission Type^3^**							
Suspect	0.73	0.76	57 (68)	2.09	0.46	9.4	0.34
IDC	Reference*		21 (42)				

OR, odds ratio; SE, standard error. *Reference group for the odds ratio. **Percent of isolates that fall within each category described and total number of isolates within that category in parenthesis (*n*). ^1^Binomial variable for the odds ratio of and a serotype isolate carrying resistant gene *bla*_TEM_ when compared to all other serotypes combined. For example, Serotype Dublin when compared to *Salmonella* belonging to any other serotype. ^2^Binomial variable for the odds ratio of a *Salmonella* isolated between 2002 and 2009 carrying resistant gene *bla*_TEM_ when compared to *Salmonella* isolated between 2010 and 2016. ^3^Binomial variable for the odds ratio of a *Salmonella* isolated from an animal suspected of salmonellosis carrying resistance gene *bla*_TEM_ when compared to a *Salmonella* isolated from an animal as part of an infectious disease control protocols (IDC). Statistically significant *p* values are in bold.

### Temporal Distribution for *bla*_CMY–2_ and *bla*_TEM_ Genes

Isolates categorized as resistant to at least one third-generation cephalosporin (3GC) via MIC testing and were positive for either *bla*_CMY–2_ or *bla*_TEM_ were analyzed by year (Table 3). Out of 242 *Salmonella* isolates, 94 (39%) were phenotypically resistant to at least one 3GC. Of these 94 3GC resistant isolates, 48% (*n* = 45) were positive for *bla*_CMY–2_ only and 50% (*n* = 47) were simultaneously positive for both *bla*_CMY–2_ and *bla*_TEM_. Two third-generation cephalosporin resistant isolates, which were negative for both *bla*_CMY–2_ and *bla*_TEM_, likely house ESBL genes not screened for by our qPCR assay.

**TABLE 3:** Annual distribution of *Salmonella* isolates positive for phenotypic resistance to a third-generation cephalosporin (**3GC**) and for presence of antimicrobial resistance genes *bla*_CMY–2_ and *bla*_TEM_.

**Year**	**Total N° isolates^1^**	**N° isolates 3GC^2^ (%)**	**N° of 3GC and *bla*_CMY–2__+_^3^**	**N° of 3GC and *bla*_TEM__+_^4^**	**% of 3GC and^5^**	
					***bla*_CMY–2_+**	***bla*_TEM_+**
2002	15	11 (73)	11	11	100%	100%
2003	9	1 (11)	1	1	100%	100%
2004	17	8 (47)	8	6	100%	75%
2005	19	12 (63)	12	4	100%	33%
2006	11	5 (45)	5	4	100%	80%
2007	43	22 (51)	22	0	100%	0%
2008	49	17 (35)	16	13	94%	76%
2009	19	1 (5)	1	0	100%	0%
2010	20	6 (30)	6	0	100%	0%
2011	5	1 (20)	1	0	100%	0%
2012	3	1 (33)	1	1	100%	100%
2013	7	2 (29)	2	1	100%	50%
2014	11	5 (45)	4	4	80%	80%
2015	5	2 (40)	2	2	100%	100%
2016	6	0 (0)	0	0	–	–
TOTAL	239	94	92	47	98%	50%

*^1^Total number of *Salmonella* isolates recovered from 9162 bovine fecal samples submitted to a Veterinary Medical Teaching Hospital (VMTH) microbiology laboratory between 2002 and 2016. ^2^Number of isolates resistant to at least one third-generation cephalosporin antimicrobial. ^3^Number of isolates resistant to at least one third-generation cephalosporin antimicrobial and positive for presence of a *bla*_CMY–2_ gene. ^4^Number of isolates resistant to at least one third-generation cephalosporin antimicrobial and positive for presence of a *bla*_TEM_ gene. ^5^Percent of isolates resistant to at least one third-generation cephalosporin antimicrobial and positive for presence of a *bla*_CMY–2_ or *bla*_TEM_ gene.*

Despite fluctuations between years, there were no significant differences between individual years (Table 3). In addition, year group (2002–2009 vs. 2010–2016) was found to have no significant effect on the odds of isolating a *bla*_CMY–2–_positive, ceftriaxone resistant isolate positive for *bla*_TEM_ (Table 2). For all years with isolates resistant to at least one 3GC, nearly all of these isolates were positive for *bla*_CMY–2_. Three years (2002, 2003, and 2012) reported 100% of third-generation cephalosporin (3GC) resistant isolates (Supplementary Table S1: Isolates 1–12, 85) were positive for both *bla*_TEM_ and *bla*_CMY–2_.

### Risk Factors for Presence of *bla*_TEM_

A logistic regression model revealed that certain serotypes of *Salmonella* and submission type impacted the odds ratio of isolating a *bla*_CMY–2_ positive, ceftriaxone resistant isolate positive for *bla*_TEM_ (Table 2). A similar analysis for calculating the odds ratio of isolating a ceftriaxone resistant isolate positive for only *bla*_CMY–2_ could not be conducted due to almost all isolates testing positive for *bla*_CMY–2._ For serotype Dublin, which accounted for 38% of all isolates, the odds ratio for testing positive for *bla*_TEM_ when compared to all other serotypes was 51.6 (95% CI: 4.01–664.03, *p* = 0.0029). For serotype Typhimurium, which accounted for 9% of all isolates, the odds ratio for testing positive for *bla*_TEM_ when compared to all other serotypes was 43.3 (95% CI: 1.76–1000, *p* = 0.0216). For serotype Newport, which accounted for 42% of all isolates, the odds ratio for testing positive for *bla*_TEM_ when compared to all other serotypes was 0.96 (95% CI: 0.087–10.5, *p* = 0.97). For isolates in year group 2002–2009 (86% of total isolates) the odds ratio of carrying *bla*_TEM_ was 0.88 when compared to *Salmonella* isolated between 2010 and 2016 (24% of total isolates) (95% CI: 0.17–4.66, *p* = 0.88). Although not significant, the odds ratio of having *bla*_TEM_ for isolates collected from animals suspected of salmonellosis (68% of total isolates) was 2.09 when compared to isolates collected as part of an infectious disease control protocol (42% of total isolates) (95% CI: 0.46–9.7, *p* = 0.34).

While the three serotypes previously mentioned accounted for a majority of the 93 3GC resistant isolates, five other serotypes were also detected (Supplementary Table S1). These serotypes are Reading (Isolate 19), Meleagridis (Isolates 46, 50, and 52), Montevideo (Isolate 69), 9,12:nonmotile (Isolate 86), and Give (Isolate 93).

### Lack of *bla*_CTX–M_ and PMQR Genes

The ESBL gene *bla*_CTX–M_ was not detected in any of the 110 NTS isolates screened. Additionally, none of the 110 NTS isolates, including two isolates phenotypically resistant to nalidixic acid, were positive for the PMQR genes screened in our assay (*oqx*A, *oqx*B, *qnr*S, *qnr*B, and *aac(6′)-lb-cr*). It should be noted that the methods used in our study did not allow for the delineation between *aac(6′)-lb-cr* and other highly similar variants like *aac(6′)-lb.* Likewise, the methods used in our study did not allow for differentiation between *bla*_CTX–M_ groups.

## Discussion

Of the 242 NTS isolates tested, 39% of isolates (*n* = 94) were phenotypically resistant to a 3GC and 98% (*n* = 92) and 50% (*n* = 47) of these resistant isolates were PCR-positive for *bla*_CMY–2_ and *bla*_TEM_, respectively (Table 3). In the United States, ceftriaxone resistant NTS has primarily been observed to carry the gene *bla*_CMY–2_ encoding the AmpC-type β-lactamase (ACBL) CMY-2 (Centers for Disease Control and Prevention, 2014). The high prevalence of *bla*_CMY–2_ in 3GC resistant NTS in our study was similarly observed in a 2007 USDA study in which 81.6% of a subsample of ceftiofur resistant *Salmonella* isolates collected from 34,000 *Salmonella* isolates from the NARMS between 1999 and 2003 were positive for *bla*_CMY–2_ (Frye and Fedorka-Cray, 2007). More recently, a 2017 study which focused primarily on NTS isolated from beef cattle fecal samples detected *bla*_CMY–2_ in 8% of 571 isolates (Mollenkopf et al., 2017). Analysis of these CMY-2-positive isolates revealed 90% homology within serotypes, highlighting the clonal dissemination of *bla*_CMY–2_ within the cattle populations sampled in this study. Future work analyzing the homology within serotypes of the 92 isolates positive for *bla*_CMY–2_ in our study may be warranted given the number of isolates within the same serotype to be positive for *bla*_CMY–2_. *bla*_CMY–2_ is a very common resistance gene present when phenotypic resistance to ceftriaxone is observed, as shown in Table 3 and observed in other studies (Laurel, 2017), and represents a potential gene to focus future diagnostic approaches to classify an isolate as resistant to ceftriaxone without the need for use of phenotypic, culture-based methods. It should be noted that the methods used in our study did not allow for the delineation between *bla*_CMY–2_ and other highly similar variants like *bla*_CMY–4_.

The β-lactamase encoding gene *bla*_TEM_ was present in 50% (*n* = 47) of 3GC resistant NTS isolates in our study and which were also simultaneously positive for *bla*_CMY–2_. TEM-1, discovered in 1965, is one of the most ubiquitous β-lactamases among *Enterobacteriales* (Paterson and Bonomo, 2005; Lachmayr et al., 2009). TEM-1 is not an ESBL and generally only degrades penicillins and the earliest developed cephalosporins. The first reported TEM-type ESBL, TEM-3, was discovered in 1989 (Sougakoff et al., 1988). With TEM variants now numbering greater than 200 and with many belonging to the ESBL subclass, a significant diversity exists within this resistance mechanism (Palzkill, 2018). A 2012 French study of 204 ESBL-producing *E. coli* isolates collected from sick cattle between 2006 and 2010 revealed only 7/204 (3.4%) expressed ESBL-type TEM-52 (Haenni et al., 2012). In the context of human medicine, the presence of TEM-type ESBLs in NTS in Bangladesh poses a public health concern (Ahmed et al., 2014). The 2014 Bangladesh study of 2120 *Salmonella* isolates from 128,000 human stool samples collected between 2005 and 2013 revealed that 88% (7/8) ceftriaxone resistant strains were positive for *bla*_TEM_. It should be noted that a limitation of our study was that all isolates positive for *bla*_TEM_ were also positive for *bla*_CMY–2_ and that the methods used in our study did not allow for the delineation between the β-lactamase gene *bla*_TEM–1_ and other highly similar ESBL variants like *bla*_TEM–52_.

In the U.S., the first reported *Enterobacteriales* carrying *bla*_CTX–M_ in dairy cattle was an *E. coli* strain in a study by Wittum et al. (2010) from Ohio in 2009. Identification of CTX-M-producing NTS in the U.S. has been relatively rare, but recent detection of such isolates in both livestock and retail chicken meat in the U.S. poses a potential threat to food safety (Wittum et al., 2012; Brown et al., 2018). None of the *Salmonella* isolates screened in our study were positive for *bla*_CTX–M_.

Our multiplex qPCR assay, while originally developed for use in an epidemiological or microbiological research setting, has potential advantages over traditional phenotypic testing common in a clinical setting. While research PCR assays tend to be low throughput and prioritize the ability to detect the lowest number of gene target copies, clinical PCR assays have additional requirements including high throughput and minimizing the chance of either false positive or negative results (Bustin et al., 2009). Frequently used in a clinical setting, phenotypic antimicrobial susceptibility testing (e.g., broth microdilution or Kirby–Bauer test) relies on multiple incubations of the microorganism and requires a minimum of 12 h (Doern, 2018). A multiplex qPCR assay could be clinically relevant when performing culture-based antibiotic resistance testing. A multiplex qPCR assay could serve as a complementary, rapid screening test for antimicrobial resistance genes while phenotypic tests are being conducted. Our qPCR assay (not including initial isolation) can be completed in about 6 h. Unlike phenotypic antimicrobial susceptibility tests, our assay requires DNA extraction and a qPCR run, but does not necessitate the bacteria to be incubated twice.

Only two serotypes were shown to significantly increase the odds ratio of isolating a *bla*_CMY–2_-positive, ceftriaxone resistant isolate also positive for *bla*_TEM_, namely Dublin and Typhimurium (Table 2). For our study, 98% of isolates resistant to ceftriaxone were also positive for *bla*_CMY–2_ (Table 3); because of that we cannot indicate causation of resistance to ceftriaxone as originating from *bla*_TEM_ or *bla*_CMY–2_ gene (the latter being the most probable). A previous study conducted on colostrum fed to dairy calves screened cephalosporin resistant *E. coli* for β-lactamase resistance genes and observed, similarly to our study, that none of the isolates were positive for *bla*_CTX–M_; they also observed that 45% and 35% of these isolates were positive for *bla*_CMY–2_ and *bla*_TEM_, respectively (Awosile et al., 2017). The higher prevalence of *bla*_TEM_ observed in our study compared to the colostrum study, in addition to increased odds for detection of *bla*_TEM_ in *S.* Dublin isolates, is of critical importance as *bla*_TEM_ has been linked to resistance to cephalosporins and various other β-lactam antibiotics; reducing the potential effective antimicrobial treatment options for infections caused by pathogens (Paterson and Bonomo, 2005).

While there is little research on the effect of serotype on the odds of isolating a ceftriaxone resistant and *bla*_TEM_-positive NTS isolate in cattle; a previous study has demonstrated both serotypes to possess high levels of ceftiofur (3GC) resistance, and the driver of resistance is most probably being driven by another antimicrobial gene (Otto et al., 2018). The most recent NARMS data of human NTS isolates revealed that 66.7% of serotype Dublin and 4.7% of serotype Typhimurium isolates were resistant to ceftriaxone. Despite being a cattle-adapted serotype, Dublin causes increased hospitalization and mortality in human infections when compared to other NTS serotypes (Harvey et al., 2017). Typhimurium is also one of the most common serotypes to cause human infection in both the US and globally (Gutema et al., 2019).

Our prior study evaluating phenotypic resistance of *Salmonella* isolates from cattle observed a 13.7 higher odds (*p* value = 0.0004) for isolating a multidrug resistant *Salmonella* from suspect clinical salmonellosis cases when compared to isolates originating from the VMTH IDC protocol sampling (Davidson et al., 2018). Our current study further evaluated specific resistance mechanisms for cephalosporin and fluoroquinolone resistance genes. We did not detect a significant difference in the odds ratio for isolating *Salmonella* from animals suspected of salmonellosis when compared to isolates originating from the IDC program for the resistance genes screened. This result could indicate that cephalosporin and fluoroquinolone resistance genes were not the main factors increasing the risk for MDR isolates between these two different sources of *Salmonella* isolates. Although antimicrobial resistance is not in itself a virulence factor, it is a key factor in development of infection, and may be considered a virulence-like factor in specific ecological niches which antibiotic resistant bacteria are able to colonize (Beceiro et al., 2013). This is especially consistent in a hospital environment where, if an opportunistic pathogen is drug resistant, it can cause disease more readily. Mutations increasing antimicrobial resistance have a range of effects on bacterial fitness during infection including decreased or increased pathogenic potential. Future studies should further elucidate the determinants of altered virulence potential in resistant pathogens and illuminate the mechanisms by which resistance traits modulate the outcome of disease in veterinary hospitals (Geisinger and Isberg, 2017). A limitation of our study was that the sample population were animals from a VMTH, and may not necessarily be extrapolated to other populations that may not be under similar circumstances and also explain a wider confidence interval for some of the variables evaluated in the model. Another limitation is that *qnr*A was not included as one of the PMQR genes screened in the qPCR assay; this was due to the very low risk of detecting qnrA in *Salmonella* of cattle origin (Carroll et al., 2017).

Out of 242 *Salmonella* isolates, 39% (*n* = 94) were resistant to at least one 3GC. Of these 3GC resistant isolates, 98% (*n* = 92) were positive for *bla*_CMY–2_ and 50% (*n* = 47) were positive for *bla*_TEM_ and *bla*_CMY–2_. The consistently high prevalence of *bla*_CMY–2_ over time in isolates resistant to ceftriaxone suggests this gene may be a potential target for rapid molecular screening to identify isolates resistant to 3GC when compared to culture-based methods. The lack of isolates positive for *bla*_CTX–M_ or PMQR genes screened suggest that the cattle population evaluated continued to be low risk group for carrier of these important resistance genes. There was also no significant association between the odds ratio of isolating a *bla*_CMY–2_-positive, ceftriaxone resistant isolate also positive for *bla*_TEM_ and the year or year-group the isolates were collected. The higher odds for NTS serotype Dublin, ceftriaxone resistant isolate being positive for *bla*_TEM_ highlight the need for continued monitoring of this important cattle host-adapted strain. Overall, our study suggests that the prevalence of resistance to cephalosporins due to ESBL and ACBL genes present in bovine NTS isolates has remained relatively constant in this hospital population in Northern California from 2002 to 2016.

## Data Availability Statement

The original contributions presented in the study are included in the article/Supplementary Material, further inquiries can be directed to the corresponding author/s.

## Author Contributions

CB, BB, EE, EH, and RP conducted laboratory testing of samples. CB, BB, and RP performed data analysis and wrote the manuscript. CB, BB, AP, MC, and RP designed the study. All authors contributed to the article and approved the submitted version.

## Conflict of Interest

The authors declare that the research was conducted in the absence of any commercial or financial relationships that could be construed as a potential conflict of interest.

## References

[B1] Ahmed D. Ud-Din A. I. Wahid S. U. Mazumder R. Nahar K. Hossain A. (2014). Emergence of bla Tem type extended-spectrum beta -lactamase producing *Salmonella* spp. in the Urban Area of Bangladesh. *Isrn Microbiol.* 2014:715310. 10.1155/2014/715310 25101188 PMC4003836

[B2] Awosile B. B. McClure J. T. Sanchez J. VanLeeuwen J. Rodriguez-Lecompte J. C. Keefe G. (2017). Short communication: extended-spectrum cephalosporin-resistant *Escherichia coli* in colostrum from New Brunswick, Canada, dairy cows harbor blaCMY-2 and blaTEM resistance genes. *J. Dairy Sci.* 100 7901–7905. 10.3168/jds.2017-12941 28780105

[B3] Beceiro A. Tomas M. Bou G. (2013). Antimicrobial resistance and virulence: a successful or deleterious association in the bacterial world? *Clin. Microbiol. Rev.* 26 185–230. 10.1128/CMR.00059-12 23554414 PMC3623377

[B4] Brown A. C. Chen J. C. Watkins L. K. F. Campbell D. Folster J. P. Tate H. (2018). CTX-M-65 extended-spectrum beta-lactamase-producing *Salmonella enterica* Serotype Infantis, United States(1). *Emerg. Infect. Dis.* 24 2284–2291. 10.3201/eid2412.180500 30457533 PMC6256390

[B5] Bustin S. A. Benes V. Garson J. A. Hellemans J. Huggett J. Kubista M. (2009). The MIQE guidelines: minimum information for publication of quantitative real-time PCR experiments. *Clin. Chem.* 55 611–622. 10.1373/clinchem.2008.112797 19246619

[B6] Carroll L. M. Wiedmann M. den Bakker H. Siler J. Warchocki S. Kent D. (2017). Whole-Genome sequencing of drug-resistant *Salmonella enterica* isolates from dairy cattle and humans in New York and Washington States reveals source and geographic associations. *Appl. Environ. Microbiol.* 83 e00140–17. 10.1128/AEM.00140-17 28389536 PMC5452826

[B7] Centers for Disease Control and Prevention (2014). *NARMS 2014 Human Isolates Surveillance Report*. Atlanta, GA: Centers for Disease Control and Prevention.

[B8] Centers for Disease Control and Prevention (2019). *Antibiotic Resistance Threats in the United States 2019*. Atlanta, GA: US Department of Health and Human Services, CDC.

[B9] Ciesielczuk H. Hornsey M. Choi V. Woodford N. Wareham D. W. (2013). Development and evaluation of a multiplex PCR for eight plasmid-mediated quinolone-resistance determinants. *J. Med. Microbiol.* 62(Pt 12), 1823–1827. 10.1099/jmm.0.064428-0 24000223

[B10] Clinical and Laboratory Standards Institute (2011). *Performance Standards for Antimicrobial Susceptibility Testing: Twenty-First Informational Supplement. CLSI Document M100-S21*. Wayne, PA: Clinical and Laboratory Standards Institute.

[B11] Clinical and Laboratory Standards Institute (2020). *Performance Standards for Antimicrobial Susceptibility Testing. M100*, 30th Edn. Wayne, PA: Clinical and Laboratory Standards Institute.

[B12] Davidson K. E. Byrne B. A. Pires A. F. A. Magdesian K. G. Pereira R. V. (2018). Antimicrobial resistance trends in fecal *Salmonella* isolates from northern California dairy cattle admitted to a veterinary teaching hospital, 2002-2016. *PLoS One* 13:e0199928. 10.1371/journal.pone.0199928 29953552 PMC6023112

[B13] Dhillon R. H. Clark J. (2012). ESBLs: a Clear and Present Danger? *Crit. Care Res. Pract.* 2012:625170. 10.1155/2012/625170 21766013 PMC3135063

[B14] Doern C. D. (2018). The slow march toward rapid phenotypic antimicrobial susceptibility testing: are we there yet? *J. Clin. Microbiol.* 56:e1999–17. 10.1128/JCM.01999-17 29436417 PMC5869850

[B15] Engel T. Slotboom B. J. van Maarseveen N. van Zwet A. A. Nabuurs-Franssen M. H. Hagen F. (2017). A multi-centre prospective evaluation of the Check-Direct ESBL Screen for BD MAX as a rapid molecular screening method for extended-spectrum beta-lactamase-producing *Enterobacteriaceae* rectal carriage. *J. Hosp. Infect.* 97 247–253. 10.1016/j.jhin.2017.07.017 28743503

[B16] Frye J. G. Fedorka-Cray P. J. (2007). Prevalence, distribution and characterisation of ceftiofur resistance in *Salmonella enterica* isolated from animals in the USA from 1999 to 2003. *Int. J. Antimicrob. Agents* 30 134–142. 10.1016/j.ijantimicag.2007.03.013 17531447

[B17] Geisinger E. Isberg R. R. (2017). Interplay between antibiotic resistance and virulence during disease promoted by multidrug-resistant bacteria. *J. Infect. Dis.* 215(Suppl.1), S9–S17. 10.1093/infdis/jiw402 28375515 PMC5853982

[B18] Gutema F. D. Agga G. E. Abdi R. D. De Zutter L. Duchateau L. Gabriel S. (2019). Prevalence and Serotype Diversity of *Salmonella* in apparently healthy cattle: systematic review and meta-analysis of published studies, 2000-2017. *Front. Vet. Sci.* 6:102. 10.3389/fvets.2019.00102 31037239 PMC6476277

[B19] Haenni M. Saras E. Métayer V. Doublet B. Cloeckaert A. Madec J.-Y. (2012). Spread of the bla TEM-52 gene is mainly ensured by IncI1/ST36 plasmids in *Escherichia coli* isolated from cattle in France. *J. Antimicrob. Chemother.* 67 2774–2776. 22815353 10.1093/jac/dks282

[B20] Harvey R. R. Friedman C. R. Crim S. M. Judd M. Barrett K. A. Tolar B. (2017). Epidemiology of *Salmonella enterica* serotype dublin infections among Humans, United States, 1968-2013. *Emerg. Infect. Dis.* 23 1493–1501. 10.3201/eid2309.170136 28820133 PMC5572876

[B21] Jacoby G. A. (2009). AmpC beta-lactamases. *Clin. Microbiol. Rev.* 22 161–182. 10.1128/CMR.00036-08 19136439 PMC2620637

[B22] Karlen Y. McNair A. Perseguers S. Mazza C. Mermod N. (2007). Statistical significance of quantitative PCR. *BMC Bioinform.* 8:131. 10.1186/1471-2105-8-131 17445280 PMC1868764

[B23] Karp B. E. Campbell D. Chen J. C. Folster J. P. Friedman C. R. (2018). Plasmid-mediated quinolone resistance in human non-typhoidal *Salmonella* infections: an emerging public health problem in the United States. *Zoonoses Public Health* 65 838–849. 10.1111/zph.12507 30027554 PMC6609094

[B24] Lachmayr K. L. Kerkhof L. J. Dirienzo A. G. Cavanaugh C. M. Ford T. E. (2009). Quantifying nonspecific TEM beta-lactamase (blaTEM) genes in a wastewater stream. *Appl. Environ. Microbiol.* 75 203–211. 10.1128/AEM.01254-08 18997031 PMC2612200

[B25] Laurel M. (2017). *The National Antimicrobial Resistance Monitoring System: NARMS Integrated Report 2015*. Silver Spring, MD: Food and Drug Administration.

[B26] Mahmud S. Nazir K. Rahman M. T. (2018). Prevalence and molecular detection of fluoroquinolone-resistant genes (qnrA and qnrS) in *Escherichia coli* isolated from healthy broiler chickens. *Vet World* 11 1720–1724. 10.14202/vetworld.2018.1720-1724 30774264 PMC6362325

[B27] Mollenkopf D. F. Mathys D. A. Dargatz D. A. Erdman M. M. Habing G. G. Daniels J. B. (2017). Genotypic and epidemiologic characterization of extended-spectrum cephalosporin resistant *Salmonella enterica* from US beef feedlots. *Prev. Vet. Med.* 146 143–149. 10.1016/j.prevetmed.2017.08.006 28992919

[B28] Otto S. J. G. Ponich K. L. Cassis R. Goertz C. Peters D. Checkley S. L. (2018). Antimicrobial resistance of bovine *Salmonella enterica* ssp. enterica isolates from the Alberta Agriculture and Forestry Disease Investigation Program (2006-2014). *Can. Vet. J.* 59 1195–1201. 30410176 PMC6190149

[B29] Palzkill T. (2018). Structural and mechanistic basis for extended-spectrum drug-resistance mutations in altering the specificity of TEM, CTX-M, and KPC β-lactamases. *Front. Mol. Biosci.* 5:16. 10.3389/fmolb.2018.00016 29527530 PMC5829062

[B30] Paterson D. L. Bonomo R. A. (2005). Extended-spectrum beta-lactamases: a clinical update. *Clin. Microbiol. Rev.* 18 657–686. 10.1128/CMR.18.4.657-686.2005 16223952 PMC1265908

[B31] Perez-Perez F. J. Hanson N. D. (2002). Detection of plasmid-mediated AmpC beta-lactamase genes in clinical isolates by using multiplex PCR. *J. Clin. Microbiol.* 40 2153–2162. 10.1128/jcm.40.6.2153-2162.2002 12037080 PMC130804

[B32] Polsfuss S. Bloemberg G. V. Giger J. Meyer V. Bottger E. C. Hombach M. (2011). Practical approach for reliable detection of AmpC beta-lactamase-producing *Enterobacteriaceae*. *J. Clin. Microbiol.* 49 2798–2803. 10.1128/JCM.00404-11 21632895 PMC3147735

[B33] Rawat D. Nair D. (2010). Extended-spectrum β-lactamases in gram negative bacteria. *J. Global Infect. Dis.* 2:263. 10.4103/0974-777X.68531 PMC2946684 20927289

[B34] Rodriguez-Martinez J. M. Machuca J. Cano M. E. Calvo J. Martinez-Martinez L. Pascual A. (2016). Plasmid-mediated quinolone resistance: two decades on. *Drug Resist. Updat.* 29 13–29. 10.1016/j.drup.2016.09.001 27912841

[B35] Roschanski N. Fischer J. Guerra B. Roesler U. (2014). Development of a multiplex real-time PCR for the rapid detection of the predominant beta-lactamase genes CTX-M, SHV, TEM and CIT-type AmpCs in *Enterobacteriaceae*. *PLoS One* 9:e100956. 10.1371/journal.pone.0100956 25033234 PMC4102473

[B36] Schmidt C. W. (2012). FDA proposes to ban cephalosporins from livestock feed. *Environ. Health Perspect.* 120:A106. 10.1289/ehp.120-a106 22382071 PMC3295364

[B37] Sougakoff W. Goussard S. Courvalin P. (1988). The TEM-3 β-lactamase, which hydrolyzes broad-spectrum cephalosporins, is derived from the TEM-2 penicillinase by two amino acid substitutions. *FEMS Microbiol. Lett.* 56 343–348.

[B38] Stanaway J. D. Parisi A. Sarkar K. Blacker B. F. Reiner R. C. Hay S. I. (2019). The global burden of non-typhoidal *salmonella* invasive disease: a systematic analysis for the Global Burden of Disease Study 2017. *Lancet Infect. Dis.* 19 1312–1324. 31562022 10.1016/S1473-3099(19)30418-9 PMC6892270

[B39] Strahilevitz J. Jacoby G. A. Hooper D. C. Robicsek A. (2009). Plasmid-mediated quinolone resistance: a multifaceted threat. *Clin. Microbiol. Rev.* 22 664–689. 10.1128/CMR.00016-09 19822894 PMC2772364

[B40] Tamma P. D. Doi Y. Bonomo R. A. Johnson J. K. Simner P. J. (2019). Antibacterial resistance leadership G. A primer on AmpC beta-lactamases: necessary knowledge for an increasingly multidrug-resistant world. *Clin. Infect. Dis.* 69 1446–1455. 10.1093/cid/ciz173 30838380 PMC6763639

[B41] The European Committee on Antimicrobial Susceptibility Testing (2013). *EUCAST Guidelines for Detection of Resistance Mechanisms and Specific Resistances of Clinical and/or Epidemiological Importance*. Basel: EUCAST.

[B42] Thomson K. S. (2001). Controversies about extended-spectrum and AmpC beta-lactamases. *Emerg. Infect. Dis.* 7 333–336. 10.3201/eid0702.010238 11294735 PMC2631719

[B43] Tyson G. H. Tate H. P. Zhao S. Li C. Dessai U. Simmons M. (2017). Identification of plasmid-mediated quinolone resistance in *Salmonella* Isolated from Swine Ceca and Retail Pork Chops in the United States. *Antimicrob. Agents Chemother.* 61 e1318–17. 10.1128/AAC.01318-17 28784677 PMC5610501

[B44] Wittum T. E. Mollenkopf D. F. Daniels J. B. Parkinson A. E. Mathews J. L. Fry P. R. (2010). CTX-M-type extended-spectrum beta-lactamases present in *Escherichia coli* from the feces of cattle in Ohio, United States. *Foodborne Pathog. Dis.* 7 1575–1579. 10.1089/fpd.2010.0615 20707724

[B45] Wittum T. E. Mollenkopf D. F. Erdman M. M. (2012). Detection of *Salmonella enterica* isolates producing CTX-M Cephalosporinase in U.S. livestock populations. *Appl. Environ. Microbiol.* 78 7487–7491. 10.1128/AEM.01682-12 22885753 PMC3457112

