# Supplementary material for: Detection of Cephalosporin and Fluoroquinolone Resistance Genes via Novel Multiplex qPCR in Fecal Salmonella Isolates From Northern Californian Dairy Cattle, 2002–2016
Source: Front Microbiol. 2021 Feb 15;12:601924. doi: 10.3389/fmicb.2021.601924 (PMC7917062; doi:10.3389/fmicb.2021.601924)
Supplement: Supplementary file 1 [file Data_Sheet_1.docx]

**Table S1.** Descriptive information for *Salmonella* isolates with third-generation cephalosporin resistance (n = 94) from cattle selected for qPCR testing of resistance genes in this study

| N | Year^1^ | Serotype | Submission Type^2^ | Gender | Age Group | Antibiotic Resistances ^3^ | *bla*_TEM_ only | *bla*_TEM_ + *bla*_CMY-2_ | *bla*_CMY-2_ only |
| --- | --- | --- | --- | --- | --- | --- | --- | --- | --- |
| 1 | 2002 | *S.*DUBLIN | Disease | Female | Calf | AmxAmpFoxCtfCroChlGenStrTet | 0 | 1 | 0 |
| 2 | 2002 | *S.*DUBLIN | IDC | Female | Adult | AmxAmpFoxCtfCroChlGenStrTet | 0 | 1 | 0 |
| 3 | 2002 | *S.* DUBLIN | Disease | Female | Adult | AmxAmpFoxCtfCroChlGenStrTet | 0 | 1 | 0 |
| 4 | 2002 | *S.* DUBLIN | Disease | Male | Calf | AmxAmpFoxCtfCroChlGenStrTet | 0 | 1 | 0 |
| 5 | 2002 | *S.* DUBLIN | Disease | Female | Calf | AmxAmpFoxCtfCroChlGenStrTet | 0 | 1 | 0 |
| 6 | 2002 | *S.* DUBLIN | Disease | Female | Adult | AmxAmpFoxCtfCroChlGenStrTet | 0 | 1 | 0 |
| 7 | 2002 | *S.* DUBLIN | Disease | Female | Calf | AmxAmpFoxCtfCroChlGenStrTet | 0 | 1 | 0 |
| 8 | 2002 | *S.* DUBLIN | Disease | Female | Calf | AmxAmpFoxCtfCroChlGenStrTet | 0 | 1 | 0 |
| 9 | 2002 | *S.*TYPHIMURIUM | Disease | Male | Calf | AmxAmpFoxCtfCroChlStrTet | 0 | 1 | 0 |
| 10 | 2002 | *S.* DUBLIN | Disease | Female | Calf | AmxAmpFoxCtfCroChlStrTet | 0 | 1 | 0 |
| 11 | 2002 | *S.* DUBLIN | Disease | Male | Calf | AmxAmpFoxCtfCroChlStrTet | 0 | 1 | 0 |
| 12 | 2003 | *S.*NEWPORT | Disease | Female | Adult | AmxAmpFoxCtfCroChlStrTet | 0 | 1 | 0 |
| 13 | 2004 | *S.* DUBLIN | Disease | Female | Calf | AmxAmpFoxCtfCroChlGenStrTet | 0 | 1 | 0 |
| 14 | 2004 | *S.*DUBLIN | Disease | Female | Calf | AmxAmpFoxCtfCroChlGenStrTet | 0 | 1 | 0 |
| 15 | 2004 | *S.*DUBLIN | Disease | Female | Calf | AmxAmpFoxCtfCroChlGenStrTet | 0 | 1 | 0 |
| 16 | 2004 | *S.*DUBLIN | Disease | Female | Calf | AmxAmpFoxCtfCroChlGenStrTet | 0 | 1 | 0 |
| 17 | 2004 | *S.*DUBLIN | Disease | Female | Calf | AmxAmpFoxCtfCroChlGenStrTet | 0 | 1 | 0 |
| 18 | 2004 | *S.*DUBLIN | Disease | Male | Adult | AmxAmpFoxCtfCroChlGenStrTet | 0 | 1 | 0 |
| 19 | 2004 | *S.*READING | Disease | Female | Adult | AmxAmpFoxCtfCroChlStrTet | 0 | 0 | 1 |
| 20 | 2004 | *S.*DUBLIN | Disease | Female | Calf | AmxAmpFoxCtfCroStr | 0 | 0 | 1 |
| 21 | 2005 | *S.*DUBLIN | Disease | Female | Adult | AmxAmpFoxCtfCroChlGenStrTet | 0 | 1 | 0 |
| 22 | 2005 | *S.*DUBLIN | Disease | Female | Calf | AmxAmpFoxCtfCroChlGenStrTet | 0 | 1 | 0 |
| 23 | 2005 | *S.*DUBLIN | Disease | Male | Calf | AmxAmpFoxCtfCroChlGenStrTet | 0 | 0 | 1 |
| 24 | 2005 | *S.*DUBLIN | Disease | Female | Calf | AmxAmpFoxCtfCroChlGenTet | 0 | 1 | 0 |
| 25 | 2005 | *S.*NEWPORT | Disease | Female | Adult | AmxAmpFoxCtfCroStrTet | 0 | 1 | 0 |
| 26 | 2005 | *S.*NEWPORT | Disease | Male | Calf | AmxAmpFoxCtfCroStrTet | 0 | 0 | 1 |
| 27 | 2005 | *S.*NEWPORT | Disease | Female | Adult | AmxAmpFoxCtfCroStrTet | 0 | 0 | 1 |
| 28 | 2005 | *S.*NEWPORT | Disease | Male | Calf | AmxAmpFoxCtfCroStrTet | 0 | 0 | 1 |
| 29 | 2005 | *S.*NEWPORT | Disease | Female | Adult | AmxAmpFoxCtfCroStrTet | 0 | 0 | 1 |
| 30 | 2005 | *S.*NEWPORT | Disease | Male | Adult | AmxAmpFoxCtfCroStrTet | 0 | 0 | 1 |
| 31 | 2005 | *S.*NEWPORT | IDC | Female | Adult | AmxAmpFoxCtfCroStrTet | 0 | 0 | 1 |
| 32 | 2005 | *S.*NEWPORT | IDC | Female | Adult | AmxAmpFoxCtfCroStrTet | 0 | 0 | 1 |
| 33 | 2006 | *S.*NEWPORT | IDC | Female | Adult | AmxAmpFoxCtfCroChlStrTet | 0 | 1 | 0 |
| 34 | 2006 | *S.*NEWPORT | IDC | Male | Adult | AmxAmpFoxCtfCroChlStrTet | 0 | 1 | 0 |
| 35 | 2006 | *S.*NEWPORT | Disease | Female | Adult | AmxAmpFoxCtfCroChlStrTet | 0 | 1 | 0 |
| 36 | 2006 | *S.*NEWPORT | IDC | Female | Adult | AmxAmpFoxCtfCroChlStrTet | 0 | 1 | 0 |
| 37 | 2006 | *S.*NEWPORT | Disease | Female | Adult | AmxAmpFoxCtfCroStrTet | 0 | 0 | 1 |
| 38 | 2007 | *S.*NEWPORT | Disease | Female | Calf | AmxAmpFoxCtfCroChlStrTet | 0 | 0 | 1 |
| 39 | 2007 | *S.*NEWPORT | IDC | Female | Adult | AmxAmpFoxCtfCroChlStrTet | 0 | 0 | 1 |
| 40 | 2007 | *S.*NEWPORT | Disease | Female | Calf | AmxAmpFoxCtfCroChlStrTet | 0 | 0 | 1 |
| 41 | 2007 | *S.*NEWPORT | IDC | Female | Adult | AmxAmpFoxCtfCroChlStrTet | 0 | 0 | 1 |
| 42 | 2007 | *S.*NEWPORT | IDC | Female | Adult | AmxAmpFoxCtfCroChlStrTet | 0 | 0 | 1 |
| 43 | 2007 | *S.*NEWPORT | IDC | Female | Adult | AmxAmpFoxCtfCroChlStrTet | 0 | 0 | 1 |
| 44 | 2007 | *S.*NEWPORT | IDC | Female | Adult | AmxAmpFoxCtfCroChlStrTet | 0 | 0 | 1 |
| 45 | 2007 | *S.*NEWPORT | IDC | Female | Adult | AmxAmpFoxCtfCroChlStrTet | 0 | 0 | 1 |
| 46 | 2007 | *S.*MELEAGRIDIS | IDC | Female | Calf | AmxAmpFoxCtfCroChlStrTet | 0 | 0 | 1 |
| 47 | 2007 | *S.*NEWPORT | IDC | Female | Adult | AmxAmpFoxCtfCroChlStrTet | 0 | 0 | 1 |
| 48 | 2007 | *S.*NEWPORT | Disease | Female | Adult | AmxAmpFoxCtfCroChlStrTet | 0 | 0 | 1 |
| 49 | 2007 | *S.*NEWPORT | IDC | Female | Calf | AmxAmpFoxCtfCroChlStrTet | 0 | 0 | 1 |
| 50 | 2007 | *S.*MELEAGRIDIS | IDC | Female | Calf | AmxAmpFoxCtfCroChlStrTet | 0 | 0 | 1 |
| 51 | 2007 | *S.*NEWPORT | Disease | Female | Adult | AmxAmpFoxCtfCroChlStrTet | 0 | 0 | 1 |
| 52 | 2007 | *S.*MELEAGRIDIS | Disease | Female | Adult | AmxAmpFoxCtfCroChlStrTet | 0 | 0 | 1 |
| 53 | 2007 | *S.*NEWPORT | Disease | Female | Adult | AmxAmpFoxCtfCroChlStrTet | 0 | 0 | 1 |
| 54 | 2007 | *S.*NEWPORT | IDC | Female | Adult | AmxAmpFoxCtfCroChlStrTet | 0 | 0 | 1 |
| 55 | 2007 | *S.*NEWPORT | Disease | Male | Calf | AmxAmpFoxCtfCroChlStrTet | 0 | 0 | 1 |
| 56 | 2007 | *S.*NEWPORT | IDC | Female | Adult | AmxAmpFoxCtfCroChlStrTet | 0 | 0 | 1 |
| 57 | 2007 | *S.*NEWPORT | Disease | Female | Adult | AmxAmpFoxCtfCroChlStrTet | 0 | 0 | 1 |
| 58 | 2007 | *S.*NEWPORT | IDC | Female | Adult | AmxAmpFoxCtfCroChlStrTet | 0 | 0 | 1 |
| 59 | 2007 | *S.*NEWPORT | IDC | Female | Adult | AmxAmpFoxCtfCroStrTet | 0 | 0 | 1 |
| 60 | 2008 | *S.*DUBLIN | Disease | Female | Calf | AmxAmpFoxCtfCroChlGenStrTet | 0 | 1 | 0 |
| 61 | 2008 | *S.*DUBLIN | Disease | Female | Calf | AmxAmpFoxCtfCroChlGenStrTet | 0 | 1 | 0 |
| 62 | 2008 | *S.*DUBLIN | Disease | Female | Calf | AmxAmpFoxCtfCroChlGenStrTet | 0 | 1 | 0 |
| 63 | 2008 | *S.*DUBLIN | Disease | Female | Calf | AmxAmpFoxCtfCroChlGenStrTet | 0 | 1 | 0 |
| 64 | 2008 | *S.*DUBLIN | Disease | Female | Adult | AmxAmpFoxCtfCroChlGenStrTet | 0 | 1 | 0 |
| 65 | 2008 | *S.*DUBLIN | IDC | Female | Adult | AmxAmpFoxCtfCroChlGenStrTet | 0 | 0 | 1 |
| 66 | 2008 | *S.*DUBLIN | IDC | Female | Adult | AmxAmpFoxCtfCroChlGenTet | 0 | 1 | 0 |
| 67 | 2008 | *S.*DUBLIN | Disease | Female | Calf | AmxAmpFoxCtfCroChlGenTet | 0 | 1 | 0 |
| 68 | 2008 | *S.*DUBLIN | Disease | Female | Calf | AmxAmpFoxCtfCroChlGenTet | 0 | 1 | 0 |
| 69 | 2008 | *S.*MONTEVIDEO | Disease | Female | Calf | AmxAmpFoxCtfCroChlGenTet | 0 | 0 | 0 |
| 70 | 2008 | *S.*TYPHIMURIUM | IDC | Male | Adult | AmxAmpFoxCtfCroChlStrTet | 0 | 1 | 0 |
| 71 | 2008 | *S.*TYPHIMURIUM | IDC | Female | Calf | AmxAmpFoxCtfCroChlStrTet | 0 | 1 | 0 |
| 72 | 2008 | *S.*DUBLIN | Disease | Female | Calf | AmxAmpFoxCtfCroChlStrTet | 0 | 1 | 0 |
| 73 | 2008 | *S.*TYPHIMURIUM | Disease | Female | Calf | AmxAmpFoxCtfCroChlStrTet | 0 | 1 | 0 |
| 74 | 2008 | *S.*DUBLIN | Disease | Female | Calf | AmxAmpFoxCtfCroChlStrTet | 0 | 1 | 0 |
| 75 | 2008 | *S.*NEWPORT | IDC | Female | Calf | AmxAmpFoxCtfCroChlStrTet | 0 | 0 | 1 |
| 76 | 2008 | *S.*NEWPORT | IDC | Female | Adult | AmxAmpFoxCtfCroChlStrTet | 0 | 0 | 1 |
| 77 | 2009 | *S.*TYPHIMURIUM | IDC | Female | Adult | AmxAmpFoxCtfCroChlStrTet | 0 | 0 | 1 |
| 78 | 2010 | *S.*NEWPORT | Disease | Female | Calf | AmxAmpFoxCtfCroChlStrTet | 0 | 0 | 1 |
| 79 | 2010 | *S.*NEWPORT | Disease | Male | Calf | AmxAmpFoxCtfCroChlStrTet | 0 | 0 | 1 |
| 80 | 2010 | *S.*NEWPORT | IDC | Female | Calf | AmxAmpFoxCtfCroChlStrTet | 0 | 0 | 1 |
| 81 | 2010 | *S.*NEWPORT | IDC | Female | Adult | AmxAmpFoxCtfCroChlStrTet | 0 | 0 | 1 |
| 82 | 2010 | *S.*NEWPORT | Disease | Female | Calf | AmxAmpFoxCtfCroChlStrTet | 0 | 0 | 1 |
| 83 | 2010 | *S.*NEWPORT | Disease | Female | Adult | AmxAmpFoxCtfCroChlStrTet | 0 | 0 | 1 |
| 84 | 2011 | *S.*NEWPORT | Disease | Female | Adult | AmxAmpFoxCtfCroChlStrTet | 0 | 0 | 1 |
| 85 | 2012 | *S.*TYPHIMURIUM | IDC | Female | Adult | AmxAmpFoxCtfCroChlStrTet | 0 | 1 | 0 |
| 86 | 2013 | *S.* SP. 9,12:NONMOTILE | Disease | Female | Calf | AmxAmpFoxCtfCroChlNalStrTet | 0 | 0 | 1 |
| 87 | 2013 | *S.*DUBLIN | Disease | Male | Calf | AmxAmpFoxCtfCroChlStrTet | 0 | 1 | 0 |
| 88 | 2014 | *S.*DUBLIN | Disease | Female | Calf | AmxAmpFoxCtfCroChlGenTet | 0 | 1 | 0 |
| 89 | 2014 | *S.*TYPHIMURIUM | IDC | Female | Adult | AmxAmpFoxCtfCroChlStrTet | 0 | 1 | 0 |
| 90 | 2014 | *S.*DUBLIN | Disease | Female | Calf | AmxAmpFoxCtfCroChlStrTet | 0 | 1 | 0 |
| 91 | 2014 | *S.*DUBLIN | Disease | Female | Adult | AmxAmpFoxCtfCroChlStrTet | 0 | 1 | 0 |
| 92 | 2014 | *S.*TYPHIMURIUM | Disease | Male | Calf | AmxAmpFoxCtfCroChlStrTet | 0 | 0 | 0 |
| 93 | 2015 | *S.*GIVE | Disease | Male | Calf | AmxAmpFoxCtfCroChlNalStrTet | 0 | 1 | 0 |
| 94 | 2015 | *S.*DUBLIN | Disease | Female | Adult | AmxAmpFoxCtfCroChlStrTet | 0 | 1 | 0 |

1. Year in which *Salmonella* was isolated from fecal sample.

2. Submitted as part of a veterinary hospital infectious disease control (IDC) program or due to animals suspected of having salmonellosis.

3. Amx: Amoxicillin, Amp: Ampicillin, Fox: Cefoxitin, Ctf: Ceftiofur, Cro: Ceftriaxone, Chl: Chloramphenicol, Nal: nalidixic acid, Gen: Gentamicin, Str: Streptomycin, and Tet: Tetracycline.

**Table S2.** Descriptive information for *Salmonella* isolates from cattle selected as controls for PCR testing. Isolates were primarily selected based on pan-susceptible phenotype, however for 2002 and 2004 no pan-susceptible isolate was available. Isolates susceptible to ceftriaxone and resistant to the lowest number of drug classes were selected. Two control isolates were selected for year 2007 because a greater number of isolates for that year was included in the study. None of the control isolates were positive for *bla_CMY-2_*.

| N | Year* | Serotype | Submission Type** | Gender | Age Group | Antibiotic Resistances *** | *bla*_TEM_ *** |
| --- | --- | --- | --- | --- | --- | --- | --- |
| 95 | 2002 | *S.*DUBLIN | IDC | Female | Adult | Str | 0 |
| 96 | 2003 | *S*. HEIDELBERG | Suspect | Female | Adult |  | 0 |
| 97 | 2004 | *S.*TYPHIMURIUM | Suspect | Female | Adult | AmpStrTet | 1 |
| 98 | 2005 | *S*. SENFTENBERG | Suspect | Female | Adult |  | 0 |
| 99 | 2006 | *S.*MONTEVIDEO | IDC | Female | Adult |  | 0 |
| 100 | 2007 | *S*. INFANTIS | Suspect | Female | Calf |  | 0 |
| 101 | 2007 | *S*. MELEAGRIDIS | IDC | Female | Adult |  | 0 |
| 102 | 2008 | *S*. LEXINGTON | IDC | Female | Adult |  | 0 |
| 103 | 2009 | *S*. UGANDA | IDC | Female | Adult |  | 0 |
| 104 | 2010 | *S*. BARRANQUILLA | IDC | Female | Adult |  | 0 |
| 105 | 2011 | *S*. MBANDAKA | IDC | Female | Adult |  | 0 |
| 106 | 2012 | *S*. ENTERIDITIS | IDC | Female | Adult |  | 0 |
| 107 | 2013 | *S.*MONTEVIDEO | IDC | Female | Calf |  | 0 |
| 108 | 2014 | *S*. UGANDA | IDC | Female | Adult |  | 0 |
| 109 | 2015 | *S.*MONTEVIDEO | IDC | Female | Adult |  | 0 |
| 110 | 2016 | *S.*MONTEVIDEO | Suspect | Female | Calf |  | 0 |

* Year in which *Salmonella* was isolated from fecal sample

** Submitted as part of a Veterinary Medical Teaching Hospital Infectious Disease Control (IDC) program or due to animals suspected of having salmonellosis.

*** Amp: Ampicillin, Str: Streptomycin, and Tet: Tetracycline.

**** Isolates positive for only *bla*_TEM_.

**Table S3**. Efficiencies, y-intercepts, and sensitivities of singleplex and multiplex assays using Universal Master Mix (Applied Biosystems). *qnr*S was tested separately via singleplex qPCR.

Sensitivity log (Sl) = (40-y intercept)/S. Sensitivity copy number (CN) = 10^Sl^.

|  | Singleplex (Universal MM) | | | Multiplex (Gene Expression MM) | | |
| --- | --- | --- | --- | --- | --- | --- |
| Gene | **Efficiency** | **Y-intercept** | **Sensitivity** | **Efficiency** | **Y-intercept** | **Sensitivity** |

| *bla*_TEM_ | **97%** | **37.2** | **<10** | **91.3%** | **39.5** | **<10** |
| --- | --- | --- | --- | --- | --- | --- |
| *oqx*A | **95.7%** | **36.99** | **<10** | **92.3%** | **38.2** | **<10** |
| *qnr*S | **93.8%** | **37.7** | **<10** |  |  |  |
| *bla*_CTX-M_ | **92.3%** | **35.5** | **<100** | **99.3%** | **39.7** | **<10** |
| *bla*_CMY-2_ | **97.2%** | **37.9** | **<10** | **95.7%** | **38.3** | **<10** |
| *oqx*B | **93.8%** | **37.3** | **<10** | **96.5%** | **38.4** | **<10** |
| *aac(6’)-lb-cr* | **99.7%** | **38.5** | **<10** | **99.7%** | **38.5** | **<10** |
| *qnr*B | **98.4%** | **38.3** | **<10** | **94.2%** | **37.2** | **<10** |
